# Wrist posture unpredictably affects perception of targeted transcutaneous electrical nerve stimulation with wrist-placed electrodes

**DOI:** 10.3389/fnins.2024.1490828

**Published:** 2024-12-09

**Authors:** Neha Thomas, Luke Osborn, Courtney Moran, Matthew Fifer, Breanne Christie

**Affiliations:** Research and Exploratory Development Department, Johns Hopkins University Applied Physics Laboratory, Laurel, MD, United States

**Keywords:** electrical stimulation, human, haptic feedback, peripheral nerve stimulation, perception

## Abstract

**Objective:**

Targeted transcutaneous electrical nerve stimulation (tTENS) is a non-invasive neural stimulation technique that involves activating sensory nerve fibers to elicit tactile sensations in a distal, or referred, location. Though tTENS is a promising approach for delivering haptic feedback in virtual reality or for use by those with somatosensory deficits, it was not known how the perception of tTENS might be influenced by changing wrist position during sensorimotor tasks.

**Approach:**

We worked with 12 able-bodied individuals and delivered tTENS by placing electrodes on the wrist, thus targeting the ulnar, median, and radial nerves, and eliciting tactile sensations in the hand. We recorded perceptual data across three wrist postures: neutral, 45° extension, and 45° flexion. For each posture, the participants drew where they perceived the elicited percepts on a map of the hand. They verbally reported the quality of the percepts in their own words. We also varied the pulse amplitude and width of the stimulation to generate a strength-duration curve, from which we extracted the rheobase current and chronaxie time. Linear mixed models were run on the slope and intercept of the linear fit between pulse width and pulse amplitude to investigate effects of gender, posture, and electrode placement.

**Main results:**

As wrist posture changed, sensation quality was modulated for half of the participants, and percept location changed for 11/12 participants. The rheobase, chronaxie, and percept sizes were influenced by wrist posture, but the direction of these changes varied by participant and therefore the effect was not systematic. The statistical models indicated interactions between posture and electrode placement, as well as an effect of gender.

**Significance:**

If using tTENS with electrodes placed on the wrist to convey haptic feedback during sensorimotor tasks, in which wrist posture will likely change, it may be important to characterize perception on an individual basis.

## Introduction

Haptic feedback is critical for manipulating objects (Johansson and Flanagan, 2009) and plays a major role in emotional connection (Eid and Al, 2016). The role of cutaneous touch is especially important when visual feedback is unavailable or unreliable (Ernst and Banks, 2002). For those living with somatosensory deficits in their hands, it can be difficult to grip objects with an appropriate amount of force that will neither crush the object nor allow it to slip from one’s grasp. With current technological advancements, there are also many scenarios in which we do not have haptic feedback but may benefit from it, such as in telemedicine or in virtual reality.

Haptic feedback can be administered via invasive or non-invasive neural interfaces. Electrical stimulation can be delivered at various points along the somatosensory pathway to elicit tactile percepts, or “artificial touch,” which can be used as a source of haptic feedback. Prior studies have delivered electrical stimulation to the somatosensory cortex to elicit artificial touch in the hands of people with spinal cord injury (Fifer et al., 2022; Flesher et al., 2016; Armenta Salas et al., 2018; Christie et al., 2022). Other studies implanted neural interfaces onto the peripheral nerves or spinal cord to elicit artificial touch percepts in the missing limbs of people with amputations (Chandrasekaran et al., 2020; Christie et al., 2020; Raspopovic et al., 2014; Tan et al., 2014; Petrini et al., 2019; Nanivadekar et al., 2023; Davis et al., 2016). These approaches can selectively activate distinct populations of neurons, which allows them to elicit artificial touch in different regions of the hands. However, invasive neural interfaces are expensive and require surgical intervention, so they may not be appealing to many individuals, particularly when the use-case is not for health purposes.

“Smart gloves” are a non-invasive method for delivering haptic feedback. Smart gloves typically contain vibrating motors, pneumatic chambers, wire actuators, or transcutaneous electrical nerve stimulation (TENS) electrodes that are placed on a person’s hand (Sadihov et al., 2013; Zhu et al., 2020; Teslasuit, 2024). These devices are intuitive and fairly easy to use, but some gloves are bulky and constrain natural hand movements and sensations. Smart gloves that incorporate TENS electrodes tend to be less bulky than other hardware (Kourtesis et al., 2022). Commonly, TENS delivers an electrotactile stimulus that is felt in the skin directly underneath the electrode (Valle et al., 2020; Han et al., 2023; Suga et al., 2023; Garenfeld et al., 2021). Artificial touch elicited by TENS has been described as feeling like tingling, vibration, pulsing, fluttering, tapping, electricity, or touch (Collu et al., 2023; Shin et al., 2018; Sang et al., 2003). The perception of TENS obeys a strength-duration curve, in which perception is governed by an inverse relationship between pulse amplitude and pulse width/duration (Collu et al., 2023; Forst et al., 2015). As pulse amplitude increases, the pulse width needed to maintain detection of the stimulus decreases. Therefore, it is possible to scale TENS amplitude as a method for conveying haptic feedback about grip force.

A newer approach, “targeted” TENS (tTENS), involves placing electrodes proximally to target and activate the underlying sensory fibers that innervate the hand (i.e., median, ulnar, and radial nerves) to elicit tactile sensations distal to the electrode (Mesias et al., 2023; Osborn et al., 2020). tTENS can be used as an approach to deliver haptic feedback to the hand without placing constraints on the hand itself. tTENS electrodes placed on the forearm, biceps, elbow, or wrist (Collu et al., 2023; Forst et al., 2015; Geng et al., 2011; Vargas et al., 2020) have successfully elicited tactile sensations in the hands of able-bodied individuals and in the missing hands of people with upper-limb amputations (Osborn et al., 2020; D’Anna et al., 2017). In a prior study, able-bodied individuals received haptic feedback from tTENS about an object that was being picked up by a prosthetic hand (Vargas et al., 2020). tTENS amplitude was scaled with respect to fingertip force. The individuals were able to determine object shape and recognize topological surface features with high levels of accuracy. Similarly, tTENS has also been integrated with prosthetic hands to provide feedback about grip force for individuals with upper-limb amputations (Osborn et al., 2018).

Motor fiber activation during movement is known to modulate the transmission and processing of sensory signals. More specifically, active or passive movement can lead to an increase in sensory detection thresholds, a phenomenon known as sensory or tactile gating (Chapman et al., 1987; Post et al., 1994; Paalasmaa et al., 1991; Milne et al., 1988; Hecht et al., 2008; Duysens et al., 1990). Prior work has demonstrated that the detection of electrotactile stimuli is hindered during movement (Cai et al., 2023; Valette et al., 2023). It is not yet known if the perception of tTENS will be influenced by changing joint angles, which may shift the location of the targeted sensory fibers (Martínez-Payá et al., 2015). The stability of tTENS perception during dexterous movements will be critical for the utility of tTENS in real-time functional tasks. Therefore, the primary goal of this study was to characterize the perception of tTENS across different wrist postures. The results of this study will impact the use of tTENS by able-bodied people, people with somatosensory deficits in their hands, and people with distal upper-limb amputations. We predicted that wrist flexion and extension may influence detection thresholds and percept size, based on previous findings that motion affects electrotactile perception (Cai et al., 2023; Valette et al., 2023) and the potential for shifts in the location of underlying sensory fibers relative to the stimulating electrode. Furthermore, we expected changes in the perceived location of the evoked percept as different neural fibers, innervating distinct regions of the skin, may have been recruited. However, we hypothesized that the qualities of the evoked percepts would remain consistent, as the quality of touch elicited by peripheral nerve stimulation is primarily influenced by stimulation parameters (Graczyk et al., 2022).

## Methods

### Targeted TENS

This study was conducted with 12 able-bodied people (seven male) between 21 and 41 years old (30.7 ± 6.51 years, mean ± standard deviation). The study protocol was approved by the Johns Hopkins Medicine Institutional Review Board. Informed consent was obtained prior to participation in research-related activities.

Rectangular tTENS waveforms were delivered using a DS8R Biphasic Constant Current Stimulator (Digitimer®, Welwyn Garden City, UK). The electrodes were ~ 1 cm in diameter, dry, and adhered to the skin using medical tape. The stimulating electrode was placed on the right wrist, and the reference electrode was placed on the metacarpophalangeal joint of the right thumb. The primary motivation for targeting the palmar side of the wrist was due to the ease of neural activation: the sensory fibers innervating the hand are fairly superficial in this location, there is minimal fat or muscle, and accessing a more distal location of the nerve improved our targeting of sensory fibers that innervate the hand rather than arm. At the start of each session, we performed an exploration in which we slowly increased stimulation pulse amplitude while the participant moved the electrode around in a mediolateral direction on the palmar side of their wrist, while the wrist was in a neutral posture. This exploration was stopped once we identified a location at which stimulation was capable of eliciting a percept in the palmar surface of their hand. The stimulating electrode placement therefore varied by person.

### tTENS perceptions in different wrist postures

tTENS detection thresholds were estimated with a two-alternative forced-choice (2-AFC) paradigm in which the participant verbally reported which of two 0.5 s intervals contained a 200 ms tTENS stimulus. Auditory cues were used to indicate the start of each interval, with a different pitch assigned to each interval. A 3 down-1 up (3D-1 U) adaptive staircase procedure was used to estimate the thresholds (Leek, 2001). We decreased the stimulus intensity until no percept was perceived, at which point a reversal occurred and the intensity increased. Three correct responses in a row at one intensity level led to a decrease in the intensity. The intensity increased after one incorrect answer. When stimulation pulse width (PW) was held constant, the pulse amplitude (PA) was changed with a step size of 0.1 mA. When PA was held constant, PW was changed with a step size of 10 or 20 μs. The 10 μs step size was used when the PA was at a higher level, because smaller changes in PW produced greater changes in delivered charge that resulted in more noticeable changes in intensity. The detection threshold was calculated by averaging the stimulus intensity across all reversals (i.e., whenever the direction of the staircase changed). This method estimates the stimulus intensity that can be detected 79.4% of the time (Leek, 2001).

Thresholds were collected while the wrist was held in three different postures (conducted in the same order across all participants): neutral, extended at a 45° angle, and flexed at a 45° angle. For the first six participants, the participants rested their fingertips on a box to maintain this angle. For the remaining six, the participants rested their hand on a 3D-printed 45° ramp. For each posture, we collected three thresholds while holding PW constant at 100, 200, or 500 μs and three thresholds while holding PA constant at 0.75, 1, or 2 mA. All pulse width and pulse amplitude thresholds were obtained once per participant for each wrist posture. For the first three participants, we attempted to hold PA constant at 0.5 mA instead of 0.7 mA; however, we found that the pulse width had to reach closer to 1,000 μs to become detectable and it became too difficult to quickly find a threshold. All stimuli were delivered at 50 Hz to ensure that the stimulus was perceived as fused/continuous (Garenfeld et al., 2021). The participants were asked to outline where they perceived the artificial touch percept(s) on a drawing of the hand; drawings were acquired while delivering tTENS at a suprathreshold stimulation level, which was selected based on what was comfortable and easily detectable across multiple trials. The drawings were scanned and processed using the MATLAB Image Processing toolbox (MathWorks, Inc.; Natick, MA, USA), which allowed us to calculate the size of the elicited percepts. The participants were also asked to describe the quality of the percepts in their own words. Experiments lasted approximately 2 h per person.

The data points collected during threshold testing were fit to a strength-duration curve using Lapicque’s equation (Equation 1) (Collu et al., 2023; Forst et al., 2015; Geddes and Bourland, 1985; Lapique, 1909; Earley and Ortiz-Catalan, 2023). Lapicque’s equation was fit to the raw data using a nonlinear least-squares method with bisquare weighting, with starting values of b = 0.1 and c = 1,000. The R-squared goodness of fit values had an average of 0.97. In Lapicque’s equation, *b* is the rheobase current, *c* is the chronaxie time, *d* is the pulse width, and *I* is the pulse amplitude.


(1) $$I=b\left(1+\frac{c}{d}\right)$$

The rheobase corresponds to the lowest pulse amplitude that is able to elicit a detectable percept at an infinitely high pulse width. The chronaxie time is the pulse width that corresponds to twice the rheobase. The resulting curve presents a relationship between the minimum pulse amplitude and pulse width required to elicit a detectable percept. However, because we typically did not test pulse widths higher than 500 μs, the Lapicque equation’s coefficients were not representative of the approximate asymptote. Instead, we approximated that the asymptote was reached near 1,000 μs based on a prior study (Forst et al., 2015), which we deemed to be the rheobase current, and calculated the chronaxie time from there.

### Statistical methods

We ran two-tailed paired t-tests between the rheobase amplitudes in the neutral vs. extended and the neutral vs. flexed conditions. We also ran the same comparisons for the chronaxie values. Finally, we ran two-tailed one-sample t-tests on the absolute value of the differences between conditions for the sizes of the evoked percepts. *p*-values less than 0.05 were considered to be statistically significant.

Lapicque’s equation was transformed into a linear relationship by multiplying by *d*, the pulse width. This linearized form is commonly referred to as Weiss’s equation (Weiss, 1901). Thus, *b* (the rheobase current) corresponded to the slope of the line and b*c was the intercept. We ran two linear mixed models on *b* and b*c. *b* was log-transformed when running the mixed model due to non-normal residuals. Covariates of the model included unique combinations of wrist posture, electrode placement, use of the ramp (15 total conditions), and gender. Custom contrasts were applied to the condition variable to make specific comparisons. Raw *p*-values were corrected for multiple comparisons.

## Results

The electrodes were placed on the palmar side of the right wrist. The optimal electrode site for eliciting artificial touch in the hand varied among the participants: a more medial/ulnar placement (toward the pinky) was best for five individuals, a central placement was best for three individuals, and a more lateral/radial placement (towards the thumb) was best for four individuals. Some of the words that the participants used to describe tTENS percept qualities were flick, buzz, twitch, pins and needles, burst, tensing, pulse, tingling, muscle spasm, tapping, poking, stroking, pulling, swelling, and vibrating (Table 1). The qualities of the reported percepts changed as a result of changing wrist posture for half of the participants. Typically, even though different words were utilized, most participants reported that changes in quality were minor. For example, with participant AB6, tingling was reported during all wrist postures, but pins and needles were also reported when in the extended posture. Only one participant (AB7) reported a completely different sensation between the neutral and flexed postures.

**Table 1:** Linear mixed model results for comparisons of the slope of Weiss’s equation (*b*, the rheobase current), followed by *post-hoc* tests.

		Comparison (condition #1 vs. condition #2)		Estimate for coefficient log(b)	Std. error	*p-*value
		*Condition #1*	*Condition #2*			
Linear mixed model results	1	Average of log(b)	0 (Mixed Model Intercept)	−5.15	0.31	<0.001
	2	Female	Male	0.03	0.65	1.00
	3	Flexed wrist	Neutral wrist	−0.01	0.37	1.00
	4	Extended wrist	Neutral wrist	−0.17	0.37	0.65
	5	Medial electrode	Lateral electrode	1.18	0.68	0.22
	6	Medial electrode	Central electrode	−1.57	0.75	0.13
	7	Flexed wrist, Central electrode, Ramp	Neutral wrist, Central electrode	−2.52	0.84	0.01
	8	Flexed wrist, Central electrode, No Ramp	Neutral wrist, Central electrode	1.07	1.14	0.71
	9	Flexed wrist, Lateral electrode, Ramp	Neutral wrist, Lateral electrode	0.54	0.82	1.00
	10	Flexed wrist, Lateral electrode, No Ramp	Neutral wrist, Lateral electrode	−0.18	0.82	1.00
	11	Flexed wrist, Medial electrode, Ramp	Neutral wrist, Medial electrode	−0.53	0.80	1.00
	12	Extended wrist, Medial electrode, Ramp	Neutral wrist, Medial electrode	−0.86	0.80	0.29
	13	Extended wrist, Medial electrode, No Ramp	Neutral wrist, Medial electrode	1.67	0.67	0.04
	14	Extended wrist, Lateral electrode, Ramp	Neutral wrist, Lateral electrode	−0.15	0.81	1.00
	15	Extended wrist, Lateral electrode, No Ramp	Neutral wrist, Lateral electrode	−0.23	0.81	0.78
	16	Extended wrist, Central electrode, Ramp	Neutral wrist, Central electrode	−2.32	0.84	0.02
*Post-hoc* tests	17	Flexed wrist	Extended wrist	0.16	0.37	1.00
	18	Central electrode	Lateral electrode	2.76	0.80	0.003
	19	Flexed wrist, Lateral electrode, Ramp	Flexed wrist, Lateral electrode, No Ramp	0.72	1.07	1.00
	20	Flexed wrist, Central electrode, Ramp	Flexed wrist, Central electrode, No Ramp	−3.59	1.34	0.06

Shaded rows indicate statistically significant *p*-values (<0.05).

**Figure 1:**
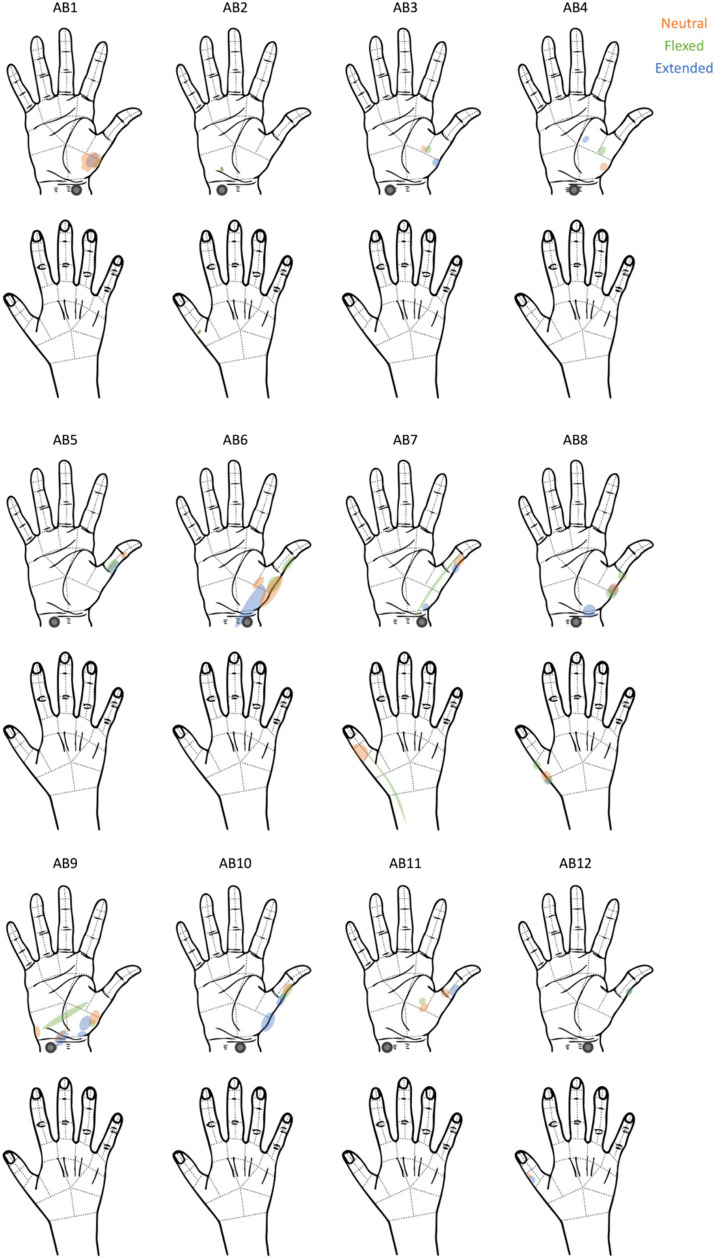
The participants’ reports of where they felt the tactile percepts elicited by tTENS. The electrodes were placed on the right wrist, as indicated by the gray circle.

**Figure 2:**
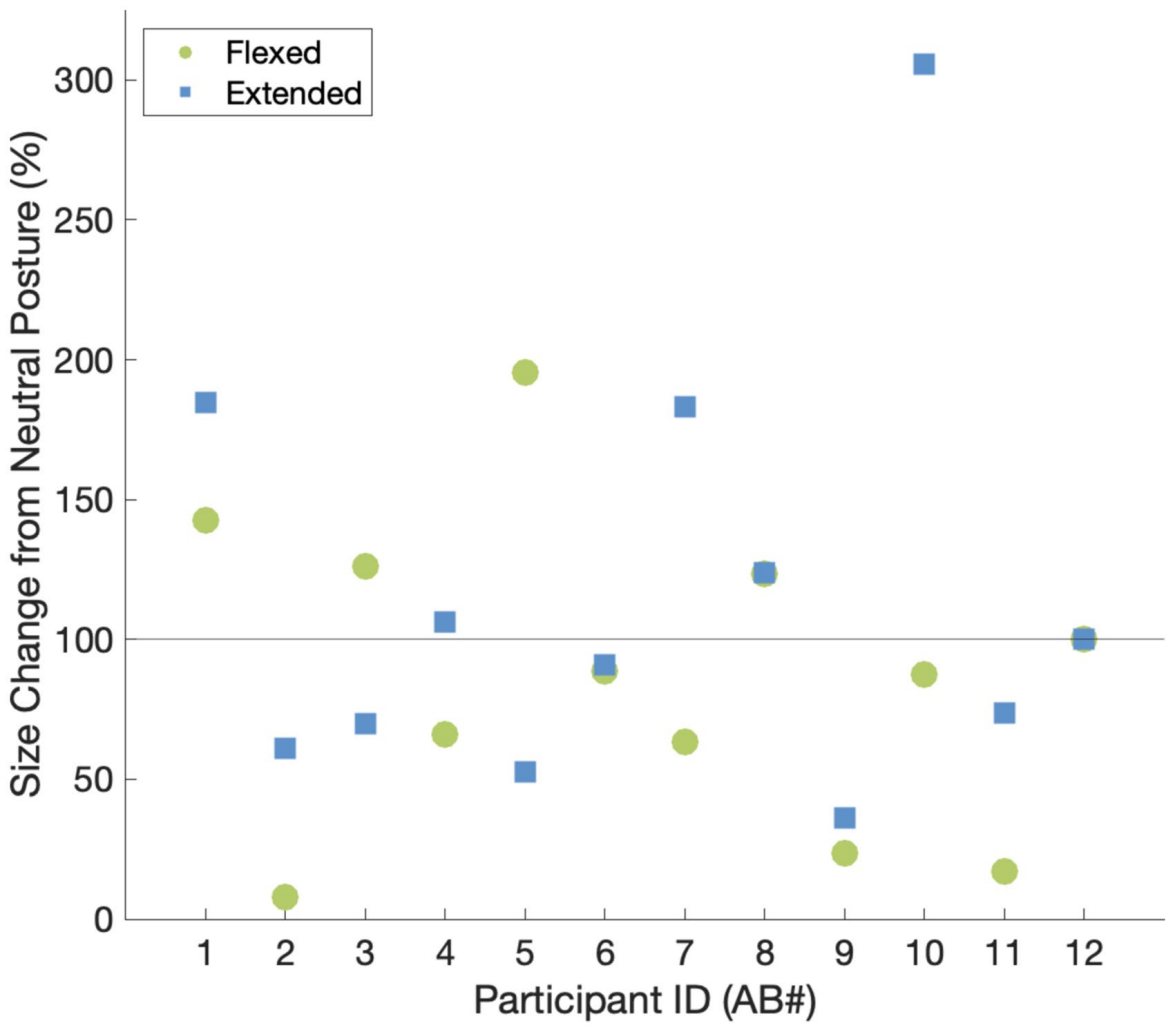
The relative changes in percept size elicited by tTENS for the flexed and extended wrist postures vs. the neutral wrist posture. A value of 100% indicates that the percept did not change in size with respect to the neutral wrist posture.

We were able to elicit tactile sensations referred to the hand in all participants (Figure 1). The locations of the reported percepts changed as a result of changing wrist posture for all participants except for AB2. The percept size was significantly different between the flexed vs. neutral condition (*p* = 0.03) and the extended vs. neutral condition (*p* = 0.01); however, the direction of change was not systematic. For instance, when moving from the neutral to the flexed wrist position, the percept sizes decreased by at least 50% for three participants and increased by ≥50% for one participant (Figure 2). Comparing the neutral to extended wrist position, percept sizes decreased by ≥50% for two participants and increased by ≥50% for three participants.

**Figure 3:**
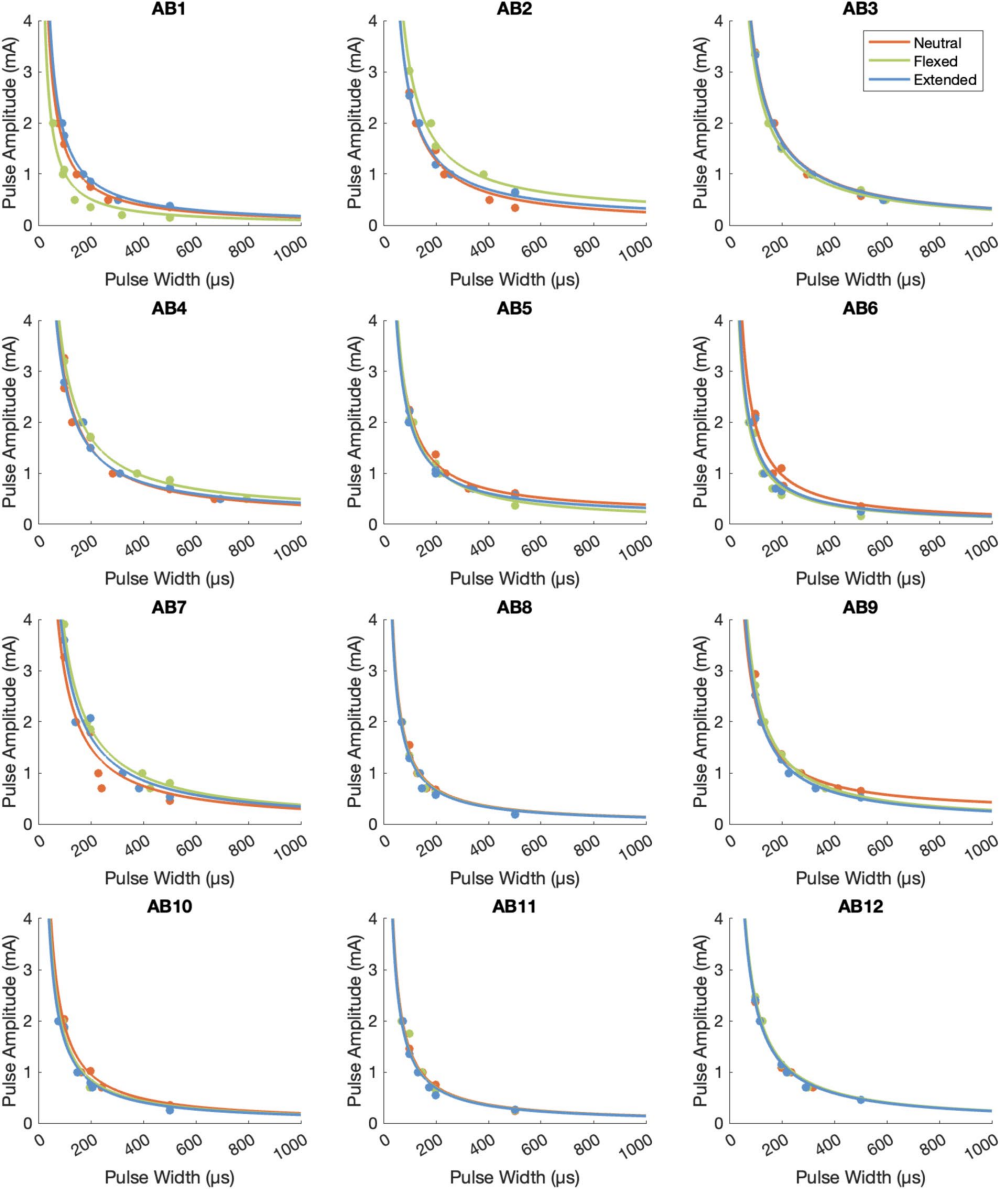
The psychometric data for tTENS perception for the 12 able-bodied participants. The raw data points are depicted with dots, and Lapicque’s equation was fit to the data from each posture.

**Figure 4:**
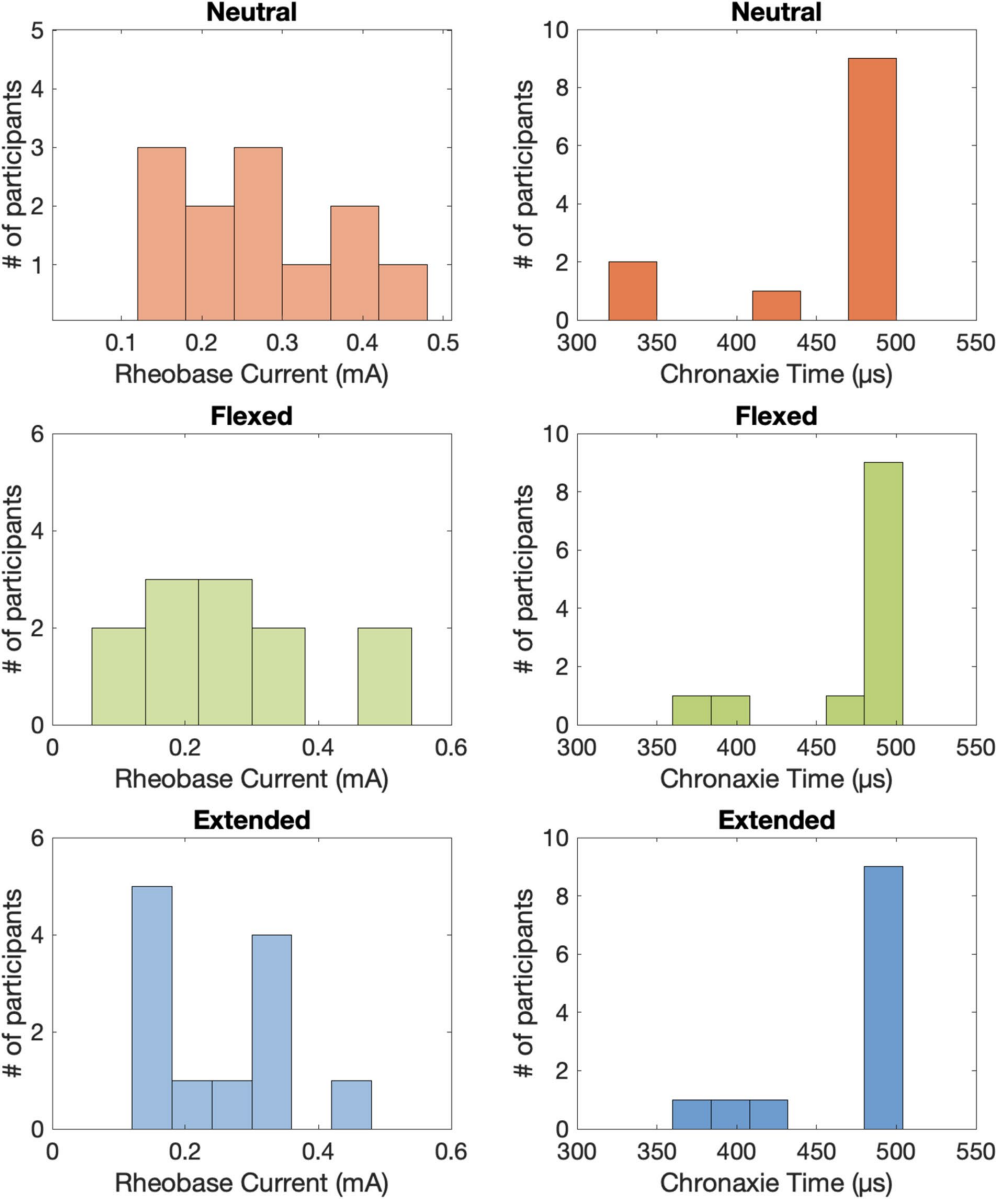
The approximated rheobase currents and chronaxie times for tTENS perception across all 12 able-bodied participants.

The psychometric data displayed an inverse relationship between pulse amplitude and pulse width (Figure 3), which is typical of a strength-duration curve (Collu et al., 2023; Forst et al., 2015). The rheobase current was 0.262 ± 0.029 mA (mean ± standard error across participants) in the neutral position, 0.258 ± 0.038 mA in the flexed position, and 0.250 ± 0.028 mA in the extended position (Figure 4). The chronaxie time was 465.37 ± 17.86 μs in the neutral position, 475.42 ± 12.77 μs in the flexed position, and 472.04 ± 13.92 μs in the extended position. The rheobase and chronaxie values were not statistically significantly different between the neutral wrist position and the flexed or extended positions (paired *t*-tests, *p* > 0.05). However, this does not mean that there were no effects of wrist posture on detection. For five participants (AB-3, 8, 10, 11, 12), the psychometric curves are nearly overlapping for the three wrist postures. However, for the remaining participants, there is some separation between the conditions, but there is not a consistent trend. As an example, for participant AB1, detection thresholds were the lowest when the wrist was in the flexed position and highest in the extended position. Conversely, for participant AB2, detection thresholds were the highest when the wrist was in the flexed position. Eight of the 12 participants had lower rheobase amplitudes (i.e., they were more sensitive) in the flexed position compared to the neutral position, though again this was not statistically significantly different. A different subset of eight participants also had lower rheobase amplitudes in the extended position compared to the neutral position.

Results of the linear-mixed models indicate that overall electrode placement on the wrist had an effect on the slope (Equation 1, *b*) of the linear relationship between pulse width and pulse amplitude, but no effect on the intercept (Equation 1, b*c) (Tables 2, 3). Central electrode placement had a steeper slope than a lateral placement. This indicates that a central electrode placement may result in higher thresholds, especially for larger pulse widths, compared to an electrode placed more laterally.

**Table 2:** Linear mixed model results for comparisons of the intercept of Weiss’s equation (b*c), followed by *post-hoc* tests.

		Comparison (condition #1 vs. condition #2)		Estimate for coefficient b*c	Std. Error	*p-*value
		*Condition #1*	*Condition #2*			
Linear mixed model results	1	Average of b*c	0 (Mixed Model Intercept)	257.08	17.9	<0.001
	2	Female	Male	−80.5	27.83	0.03
	3	Flexed wrist	Neutral wrist	4.42	6.38	0.99
	4	Extended wrist	Neutral wrist	−3.28	6.38	1.00
	5	Medial electrode	Lateral electrode	31.8	30.2	0.62
	6	Medial electrode	Central electrode	−58.7	33.4	0.21
	7	Flexed wrist, Central electrode, Ramp	Neutral wrist, Central electrode	28.6	14.7	0.13
	8	Flexed wrist, Central electrode, No Ramp	Neutral wrist, Central electrode	20.7	20.7	0.65
	9	Flexed wrist, Lateral electrode, Ramp	Neutral wrist, Lateral electrode	39.2	14.7	0.03
	10	Flexed wrist, Lateral electrode, No Ramp	Neutral wrist, Lateral electrode	−54.2	14.7	0.002
	11	Flexed wrist, Medial electrode, Ramp	Neutral wrist, Medial electrode	−17.5	14.6	0.28
	12	Extended wrist, Medial electrode, Ramp	Neutral wrist, Medial electrode	−23.7	14.6	0.23
	13	Extended wrist, Medial electrode, No Ramp	Neutral wrist, Medial electrode	−3.78	12.0	1.00
	14	Extended wrist, Lateral electrode, Ramp	Neutral wrist, Lateral electrode	22.5	14.7	0.27
	15	Extended wrist, Lateral electrode, No Ramp	Neutral wrist, Lateral electrode	−11.8	14.7	0.85
	16	Extended wrist, Central electrode, Ramp	Neutral wrist, Central electrode	13.2	14.7	0.63
*Post-hoc* tests	17	Flexed wrist	Extended wrist	7.71	6.38	1.00
	18	Central electrode	Lateral electrode	90.5	35.9	0.07
	19	Flexed wrist, Lateral electrode, Ramp	Flexed wrist, Lateral electrode, No Ramp	93.3	20.5	<0.001
	20	Flexed wrist, Central electrode, Ramp	Flexed wrist, Central electrode, No Ramp	7.94	25.2	1.00

Shaded rows indicate statistically significant *p*-values (<0.05).

**Table 3:** The participants’ reports of how tactile percepts elicited by tTENS felt.

Participant ID	Placement of stimulating electrode on the wrist	Quality, Wrist = Extended	Quality, Wrist = Flexed	Quality, Wrist = Neutral
AB1	Lateral	*Same as neutral*	*Same as neutral*	Twitch, like a fly landed on his hand
AB2	Medial	*Same as neutral*	*Same as neutral*	“Ghost twitch” pins and needles, but a single needle
AB3	Medial	*Same as neutral*	Burst pattern when stimuli became stronger	Tingling up fingers
AB4	Central	*Same as neutral*	*Same as neutral*	Tensing pulse
AB5	Medial	*Same as neutral*	*Same as neutral*	Low grade pins and needles
AB6	Lateral	Tingle, pins and needles	*Same as neutral*	Tingle
AB7	Lateral	Less abrupt sensation than neutral	Light stroke on hand	Weaker stimuli felt like buzz pulse, stronger stimuli felt like someone flicking
AB8	Central	*Same as neutral*	*Same as neutral*	Tapping
AB9	Medial	*Same as neutral*	*Same as neutral*	Rippled muscle spasm, felt in sequence (pinky, then thumb)
AB10	Central	Flick, buzz	*Same as neutral*	Flick, got longer when stimuli became stronger
AB11	Medial	Buzzy poking	Pulling, swelling	Vibrating, poking, swelling, pulling
AB12	Lateral	Pins and needles	Pins and needles	Heartbeat pulse, pins and needles

Female participants had similar slopes as male participants, but significantly lower intercepts, indicating overall lower detection thresholds. Furthermore, particular combinations of posture, electrode placement, and the use of the ramp had significant effects on the slope and intercept. A flexed posture with a central electrode with a ramp resulted in a significantly shallower slope than a neutral posture with a central electrode placement. Similarly, an extended posture with a central electrode with a ramp had a significantly shallower slope than a neutral posture with a central electrode. The intercept was significantly higher for flexed posture with lateral electrode placement with a ramp compared to without a ramp.

## Discussion

The primary objective of this study was to characterize the perception of tTENS across wrist postures that are commonly adopted during dexterous movements. Secondary objectives included investigating the effects of gender and of electrode placement. To achieve these objectives, we examined percept size, location, and quality; the chronaxie and rheobase values for each posture; and the intercept and slope of the linear fit between pulse width and pulse amplitude.

Participants reported the artificial touch percepts as originating distally in their hand rather than in the skin below the electrodes, indicating that our tTENS approach was indeed targeted and that it stimulated underlying sensory fibers that innervated the hand. Consistent with prior studies, tTENS was most commonly described as feeling like pins and needles, buzzing, and/or pulsing (Collu et al., 2023; Shin et al., 2018; Sang et al., 2003). As wrist posture changed, there were minor changes in sensation quality for half of the participants, which was contrary to our original hypothesis. However, as we predicted, there were substantial percept location changes for 11 of the 12 participants, and a statistically significant change in percept size, though the direction of change (increasing or decreasing in size) was not consistent. For the percept sizes that hovered around 100% (no change) in the extended and flexed conditions, it is possible that these small changes were actually caused by noise in drawing the percepts freehand. The participants were given a new hand map for each condition and therefore could not trace over what they drew in the other conditions. Additionally, we believe that changing wrist posture may have shifted the location of the underlying sensory fibers targeted by tTENS (Martínez-Payá et al., 2015), but that differences in individual anatomy and variations in electrode placement likely prevented there from being a systematic pattern.

There were no statistically significant differences in the rheobase or chronaxie values between the three wrist postures (neutral, extended, and flexed). While rheobase values could vary across postures, which we originally hypothesized, the effect was not systematic. Additionally, we found that placement of the stimulating electrode on the wrist significantly influenced only the slope, with a central placement resulting in steeper slopes and therefore higher thresholds. The central placement of the stimulating electrode likely targeted the median nerve for most individuals; the lower sensitivity of the central placement may be due in part to neighboring anatomical structures within the carpal tunnel that shield the nerve from the stimulus (Loh and Muraki, 2015). There was an effect of ramp usage to enforce hand posture on the results in only one experimental condition: the presence of the ramp increased the intercept for a flexed posture with a lateral electrode placement compared to without a ramp (comparison 19, Table 2).

One limitation of this study was the curve fitting of Lapicque’s equation to the raw data. Because we were not able to test pulse widths greater than 500 μs, our rheobase and chronaxie values were only estimates and not the ground truth values based on participant data. Another limitation, which would be interesting to characterize in further experiments, is that we only explored one electrode location per person. It would be interesting in future experiments to systematically vary the electrode placement on the wrist and examine the influence of stimulation location on perception. It would also be valuable to test other wrist movements, such as supination or pronation (Garenfeld et al., 2021). Although the experiment order was the same for each participant, we did not observe systematic effects of the flexed or extended posture compared to the neutral position, indicating that ordering effects and experiment duration were not major confounds.

Taken together, the results showcase the interacting effects of wrist posture, electrode placement, and rigidity of the enforced posture. The postural results are particularly relevant when the wrist is free to move. For example, for able-bodied individuals, tTENS could be used to deliver force feedback when gripping virtual objects in a virtual reality scenario, or it could be used to deliver feedback about a non-observable object characteristic (e.g., radiation level) when a person manipulates a physical object with their hands. Alternatively, tTENS could be integrated with a prosthesis as a source of haptic feedback for a person with a distal upper-limb amputation (e.g., finger-level). Overall, stimulation parameters may need to be scaled depending on posture and electrode placement to ensure consistent perception. While referred sensations in both tTENS and invasive peripheral nerve stimulation have been previously shown to be stable over several months to years (Tan et al., 2014; Osborn et al., 2020; Ackerley et al., 2018; Charkhkar et al., 2018), future research should collect more data on the stability of tTENS percepts in different wrist postures. Moreover, investigations should be conducted with varying electrode placements and arm postures, such as placing electrodes on more proximal locations (e.g., forearm, biceps) and studying the influence of elbow flexion. We anticipate that more proximal electrode locations would be less affected by wrist angle and more impacted by elbow angle, and it is possible that percepts may not be as focal. In addition, the perception of tTENS during dynamic movements should be further assessed. It is possible that while individuals actively perform a task, they may not notice minor changes in percept location, quality, or intensity/detection.

## Conclusion

In conclusion, when placing tTENS electrodes on the wrist, wrist posture impacted the perception of tactile percepts that were elicited in the hand, but there was not a consistent trend across participants. If using tTENS as tactile feedback in sensorimotor tasks, in which wrist posture will likely change, it may be important to characterize perceptual changes for individual users. Our results suggest that placing the stimulating electrode more centrally on the wrist, rather than medially or laterally, may be a more robust feedback method across multiple hand postures. Overall, though we did observe an impact of wrist posture on perception, tTENS remains a promising technique for delivering haptic feedback.

## Data Availability

The raw data supporting the conclusions of this article will be made available by the authors, without undue reservation.
